# Prognosis significance and potential association between ALDOA and AKT expression in colorectal cancer

**DOI:** 10.1038/s41598-024-57209-5

**Published:** 2024-03-18

**Authors:** Menglin Xu, Shihang Xi, Haoran Li, Yong Xia, Guangliang Mei, Zhengwu Cheng

**Affiliations:** 1 Department of Oncology, The First Affiliated Hospital of Wannan Medical College, Wuhu, 241000 China; 2 Department of Hepatobiliary Surgery, The First Affiliated Hospital of Wannan Medical College, Wuhu, 241000 China; 3 Department of Gastrointestinal Surgery, The First Affiliated Hospital of Wannan Medical College, No.2 Zheshan West Road, Jinghu, Wuhu, 241000 Anhui China; 4 Department of Education Affairs, The First Affiliated Hospital of Wannan Medical College, Wuhu, 241000 China; 5 Department of Party Affairs, The First Affiliated Hospital of Wannan Medical College, Wuhu, 241000 China

**Keywords:** Colorectal cancer, ALDOA, AKT, Prognosis, Correlation, Metastasis, Oncogenes, Tumour biomarkers

## Abstract

Colorectal cancer (CRC) is one of the most common malignant tumors of the digestive tract and a leading cause of cancer-related death worldwide. Since many CRC patients are diagnosed already in the advanced stage, and traditional chemoradiotherapy is prone to drug resistance, it is important to find new therapeutic targets. In this study, the expression levels of ALDOA and p-AKT were detected in cancer tissues and paired normal tissues, and it was found that they were significantly increased in CRC tissues, and their high expression indicated poor prognosis. Moreover, a positive correlation between the expression of ALDOA and p-AKT was found in CRC tissues and paired normal tissues. In addition, the Kaplan–Meier analysis revealed that the group with both negative of ALDOA/p-AKT expression had longer five-year survival rates compared with the other group. Besides, the group with both high expression of ALDOA/p-AKT had a worse prognosis compared with the other group. Based on the expression of ALDOA and p-AKT in tumor tissues, we can effectively distinguish tumor tissues from normal tissues through cluster analysis. Furthermore, we constructed nomograms to predict 3-year and 5-year overall survival, showing that the expression of ALDOA/p-AKT plays a crucial role in predicting the prognosis of CRC patients. Therefore, ALDOA/p-AKT may act as a crucial role in CRC, which may provide new horizons for targeted therapies for CRC.

## Introduction

Colorectal cancer (CRC) is a common malignant tumors of the digestive tract and one of the leading causes of cancer-related death worldwide^1^. Despite obvious advances in the systematic treatment of CRC, the 5-year survival rate of patients with advanced stages tumor is still poor because of distant metastases and resistance to chemotherapy^1,2^. Therefore, there is an urgent need for new therapeutic targets to improve survival and prognosis in CRC patients.

Aldolase A (ALDOA) is mainly expressed in muscle tissue. ALDOA catalyzes the reversible conversion offructose-1,6-bisphosphate to aldehyde 3-phosphate and dihydroxyacetone phosphate by encoding a glycolytic enzyme^3,4^. Studies have found that gluconeogenesis and glycolysis involved in ALDOA provide energy for the proliferation and migration in a variety of tumors, and are closely related to drug resistance of tumor cells^4,5^. The progression of tumor is accompanied by metabolic reprogramming, and ALDOA acts as an important role in tumor metabolism^6,7^. The aberrant expression of ALDOA in various tumor cells promotes the EMT process of tumor cells, enhances proliferation, and accelerates the transition from G1 to G2 phase of cell cycle by regulating tumor metabolism^8–10^.

The increased expression of ALDOA in tissues is related with the occurrence and development of various tumors. The activity of ALDOA protein in lung cancer, gastric cancer and liver cancer were significantly elevated^11–14^. Previous literatures indicated that ALDOA promotes cell proliferation and cisplatin resistance via the EGFR-AKT pathway and ALDOA expression was positively correlated with AKT expression in gastric cancer^15^. However, few study was performed to investigate the relationship between ALDOA and AKT in colorectal cancer. This study focused on the alteration of ALDOA expression in the progression of CRC and its correlation with clinical survival and prognosis. In addition, we established a prediction model, and evaluated the predictive role of ALDOA expression on survival, as well as its correlation with AKT, a target related to tumor metabolism, to provide a novel strategy for CRC targeted therapy.

## Results

### The expression of ALDOA is aberrantly increased in CRC

**Figure 1:**
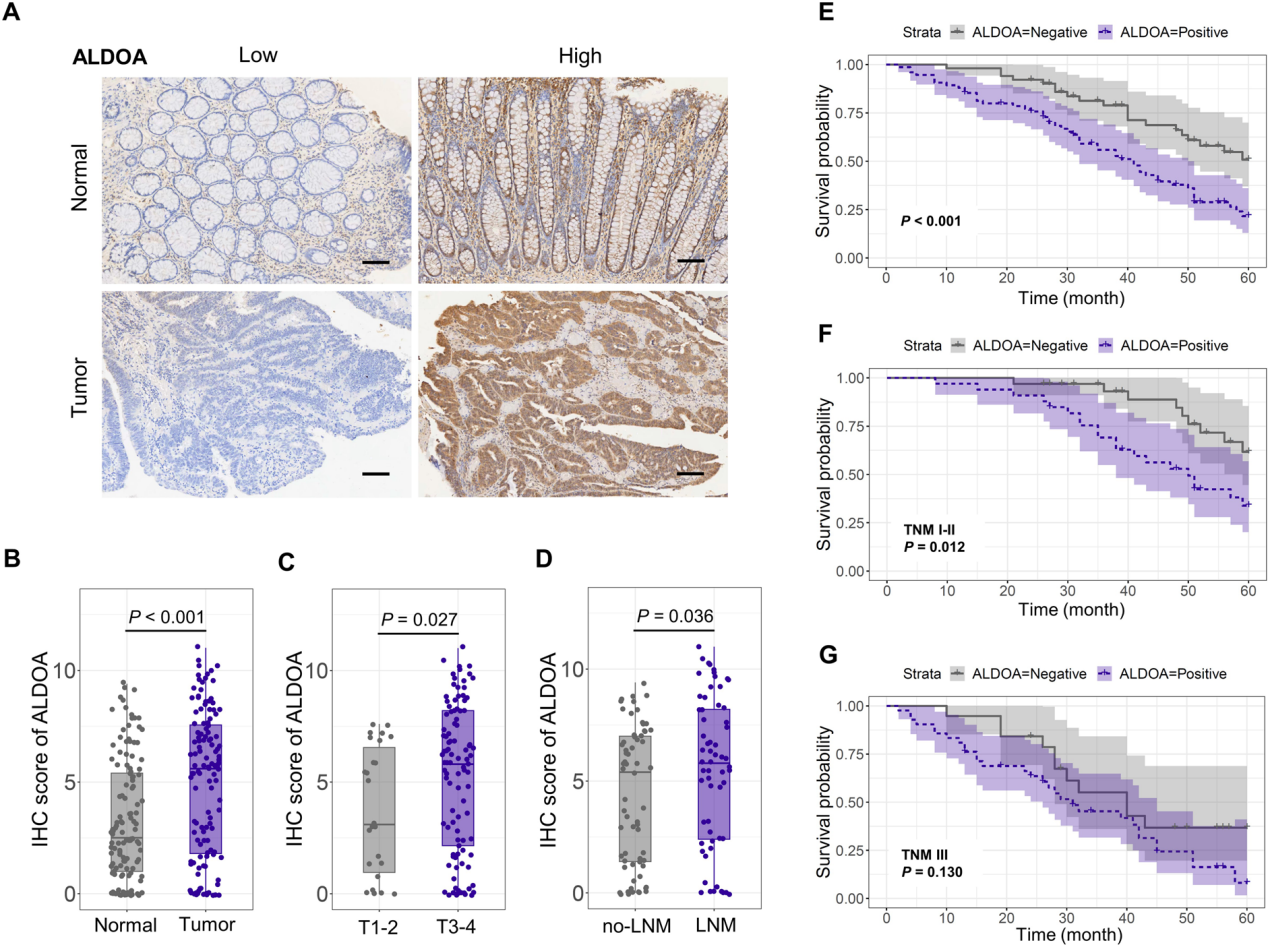
ALDOA expression increased in CRC and indicated a poor prognosis. (**A**) ALDOA expression detected by IHC staining in 126 CRC tissues and para-cancer tissues (scale bar = 100 μm). (**B**-**D**) IHC score of ALDOA in (**B**) CRC and para-cancer tissues, (**C**) CRC with T1-2 or T3-4, (**D**) CRC with or without lymph node metastasis. (**E**–**G**) Kaplan–Meier analysis of ALODA positive vs ALDOA negative in (**E**) 126 CRC patients, (**F**) the CRC patients with the TMN stage I-II, (**G**) the CRC patients with the TMN stage III.

Studies indicates that ALDOA expression is elevated in a variety of tumor tissues, but the alteration of its expression in CRC tissue and adjacent normal tissue, and its potential influence have not been fully investigated. In our study, we investigated the expression of ALDOA in 126 CRC tissues and adjacent normal tissues by IHC (Fig. 1A). The IHC score based on the staining results revealed that ALDOA expression in CRC tissues was significantly higher than that in adjacent normal tissues (Fig. 1B). In subgroup analysis, the IHC score of the T3-4 group was significantly higher than that of the T1-2 group, and the IHC score of the lymph node metastasis group was obviously higher than that of the none lymph node metastasis group (Fig. 1C,D ).

According to the IHC score, we divided the expression of ALDOA in CRC tissues and adjacent normal tissues into negative and positive. Subsequently, we evaluated the association between the ALDOA expression in CRC and clinical pathological indexes. The results revealed that the expression of ALDOA was closely related to lymph node metastasis, degree of differentiation, neural invasion and TNM stage. There is no association between ALDOA level and age, gender, tumor size, depth of tumor invasion, venous invasion (Table 1).

**Table 1:** Relationship between ALDOA and clinic-pathological factors in 126 CRC patients.

Variables	ALDOA		
	Negative	Positive	*P* value
Age (years)			
< 65	32	45	0.756
≥ 65	19	30	
Gender			
Male	28	45	0.569
Female	23	30	
Tumor size (cm)			
< 5	33	44	0.495
≥ 5	18	31	
Depth of tumor invasion			
T1-2	14	12	0.119
T3-4	37	63	
Lymph node metastasis			
No	32	33	0.039^a^
Yes	19	42	
Degree of differentiation			
Well	47	59	0.042^a^
Poor	4	16	
Venous invasion			
Negative	43	54	0.107
Positive	8	21	
Neural invasion			
Negative	45	52	0.013^a^
Positive	6	23	
TNM stage			
I-II	32	33	0.039^a^
III	19	42	

^a^*P* < 0.05.

Depending on the close connections between ALDOA and clinicopathological indicators, the influence of ALDOA expression level was further assessed in CRC tissues, affecting the overall survival rate of sufferers. Patients were classified according to ALDOA^pos^ (ALDOA positive) and ALDOA^neg^ (ALDOA negative) expression. Survival was obviously lower in patients with ALDOA^pos^ than in patients with ALDOA^neg^ (*P* < 0.001, Fig. 1E). Further subgroup analysis indicated that high ALDOA expression was closely related with a lower survival rate in the TNM stage I-II subgroup (*P* = 0.012, Fig. 1F). However, in the stage III subgroup, ALDOA expression was not statistically associated with survival (*P* = 0.130, Fig. 1G). It suggested that if the expression of ALDOA in tumor tissue is used to predict the prognosis of CRC patients, the prediction is more accurate for early CRC patients.

In addition, we performed analysis of Cox’s proportional hazard model. The univariate analysis indicated neural invasion, depth of tumor invasion, lymph node metastasis, and ALDOA expression is a prognostic factor affecting the survival of CRC patients (*P* < 0.05, Table 2). In multivariate analysis, lymph node metastasis, and ALDOA expression were independent risk factors.

**Table 2:** Results of univariate and multivariate analyses of postoperative patients’ survival by Cox’s proportional hazard model.

Varieties	Univariate analysis			Multivariate analysis		
	HR	95% CI	*P*	HR	95 % CI	*P*
Age (≤60 or >60 years)	0.880	0.547–1.418	0.600			
Gender (Male/Female)	1.177	0.731–1.895	0.502			
Size of tumor (≤5 or >5 cm)	0.813	0.507–1.303	0.390			
Degree of differentiation (moderate-well/poor)	0.565	0.314–1.017	0.057			
Venous invasion (negative/positive)	0.788	0.461–1.348	0.384			
Neural invasion (negative/positive)	0.548	0.323–0.930	0.026^a^	0.877	0.504–1.524	0.641
Depth of tumor invasion (T1-2/T3-4)	0.413	0.197–0.864	0.019^a^	0.570	0.267–1.214	0.145
Lymph node metastasis (negative/positive)	0.346	0.212–0.565	<0.001^c^	0.422	0.253–0.705	0.00^b^
ALDOA expression (negative/positive)	0.427	0.254–0.719	0.00^b^	0.496	0.292–0.841	0.009^b^

^a^*P* < 0.05, ^b^*P* < 0.01, ^c^*P* < 0.001.

### High expression of p-AKT indicates a poor prognosis in CRC

**Figure 2:**
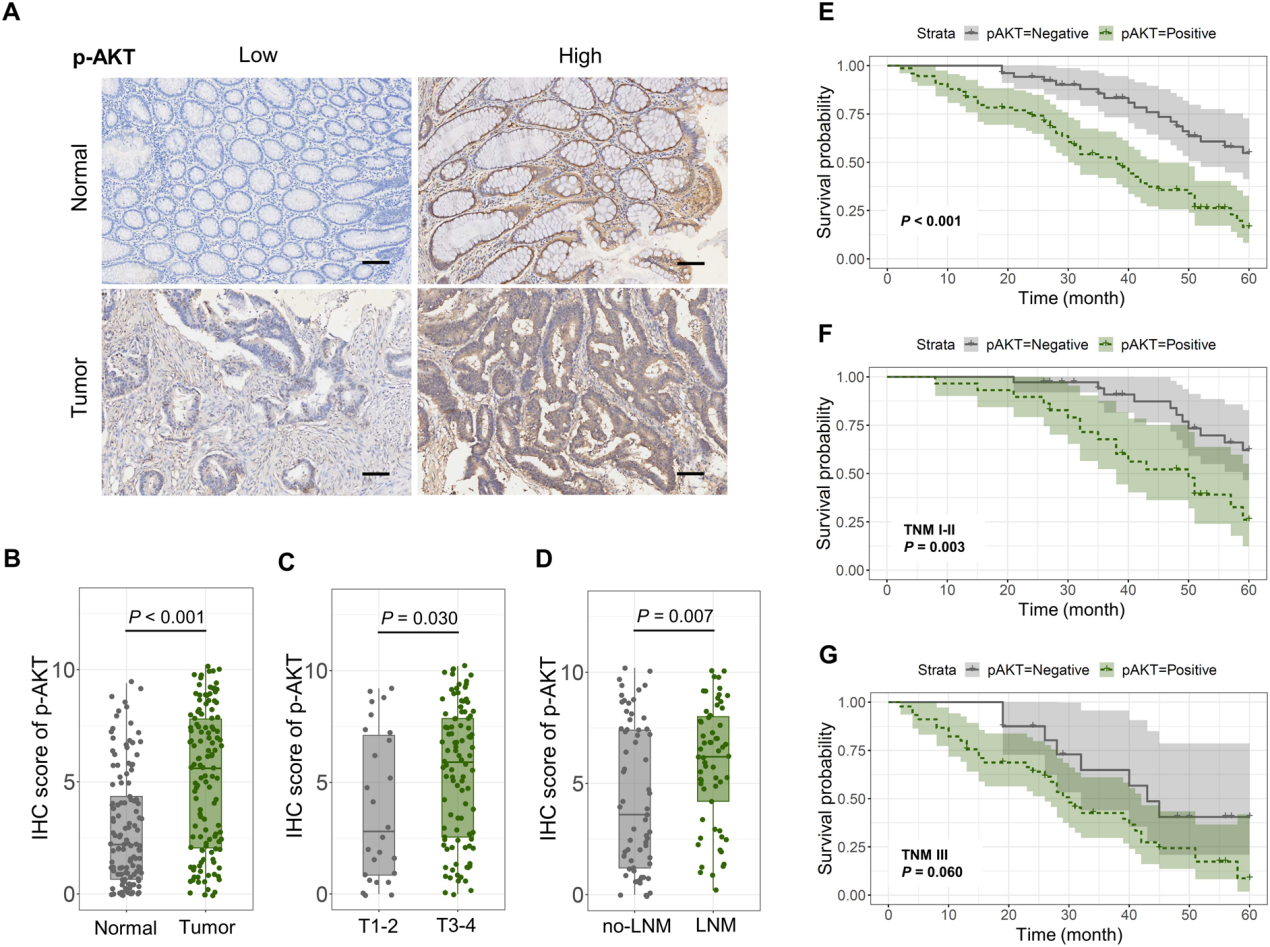
The aberrant expression of p-AKT in CRC indicated a poor prognosis. (**A**) p-AKT expression detected by IHC staining in 126 CRC tissues and para-cancer tissues (scale bar = 100 μm). (**B**-**D**) IHC score of p-AKT in (**B**) CRC and para-cancer tissues, (**C**) CRC with T1-2 or T3-4, (**D**) CRC with or without lymph node metastasis. (**E**–**G**) Kaplan–Meier analysis of p-AKT positive vs p-AKT negative in (**E**) 126 CRC patients, (**F**) the CRC patients with the TMN stage I-II, (**G**) the CRC patients with the TMN stage III.

Owing to the process of gluconeogenesis and glycolysis ALDOA included in providing energy for the proliferation and migration in cancer cells, we investigated the association between ALDOA and the expression of AKT, a classical target of energy metabolism. We also investigated the p-AKT expression in 126 CRC tissues and adjacent normal colorectal tissues by IHC, and assessed IHC scores based on the staining results (Fig. 2A). The p-AKT expression in CRC tissues was obviously higher than that in paired normal tissues (Fig. 2B). In subgroup analysis, the IHC score of the T3-4 group and lymph node metastasis group was higher than the control groups (Fig. 2C,D). Subsequently, we analyzed the effect of p-AKT expression level on survival of CRC patients. Similar to ALDOA, patients with high expression of p-AKT have a worse prognosis (Fig. 2E). For TNM stage I-II CRC patients, differences in p-AKT expression led to significant differences in prognosis (Fig. 2F). While in the TNM stage III subgroup, there was no significant correlation between p-AKT expression and postoperative survival (Fig. 2G).

### Association between ALDOA and p-AKT in CRC tissues

**Figure 3:**
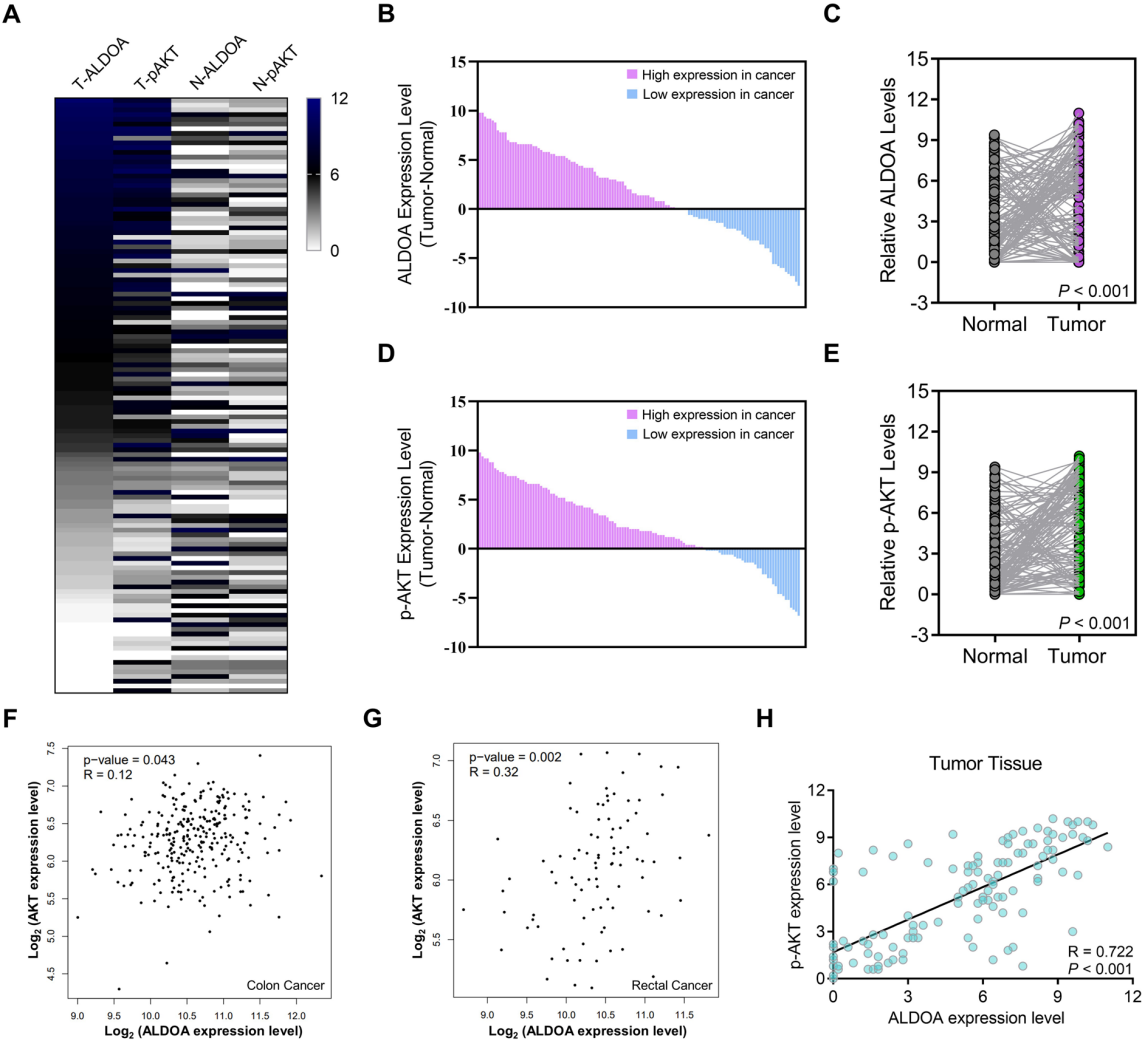
Expression of ALDOA and p-AKT in CRC and paired normal tissues. (**A**) The heatmap exhibiting the difference and correlation of expression between ALDOA and p-AKT in CRC and para-cancer normal tissues. (**B**-**C**) The alteration of ALDOA in CRC and para-cancer tissues. (**D**-**E**) The alteration of p-AKT in CRC and para-cancer tissues. (**F**-**G**) the association between *ALDOA*/*AKT* expression in TCGA datasets via GEPIA platform in (**F**) colon cancer tissues and (**G**) rectal cancer tissues. (**H**) The association between ALDOA and p-AKT expression according to IHC score in CRC tissues.

To analyze the potential correlation between ALDOA and p-AKT expression level, we showed the heatmap to exhibit the difference expression between ALDOA and p-AKT in CRC and adjacent normal tissues (Fig. 3A). The results indicated that the expression levels of ALDOA and p-AKT in tumor tissues were remarkably higher than those in normal tissues, and the expression levels of ALDOA and p-AKT were correlated to a certain extent. Subsequently, we calculated the difference of IHC score of ALDOA between CRC and paired normal tissues, and analyzed the expression of ALDOA in tumor tissue and matched normal tissue was analyzed (Fig. 3B,C). More intuitively, the expression of ALDOA in majority tumor tissues was higher than that in paired normal tissues. Then, we also analyzed the expression of p-AKT in CRC tissues and normal tissues in pairs, and the results were similar to ALDOA (Fig. 3D,E). The association between ALDOA/AKT expression in colon cancer tissues and rectal cancer tissues in TCGA datasets was investigated via GEPIA platform (Fig. 3F,G). Based on the IHC staining score, we also conducted a linear analysis of the expression of ALDOA and p-AKT in CRC tissues, and the results showed a positive correlation between them (*P* < 0.001, Fig. 3H).

**Figure 4:**
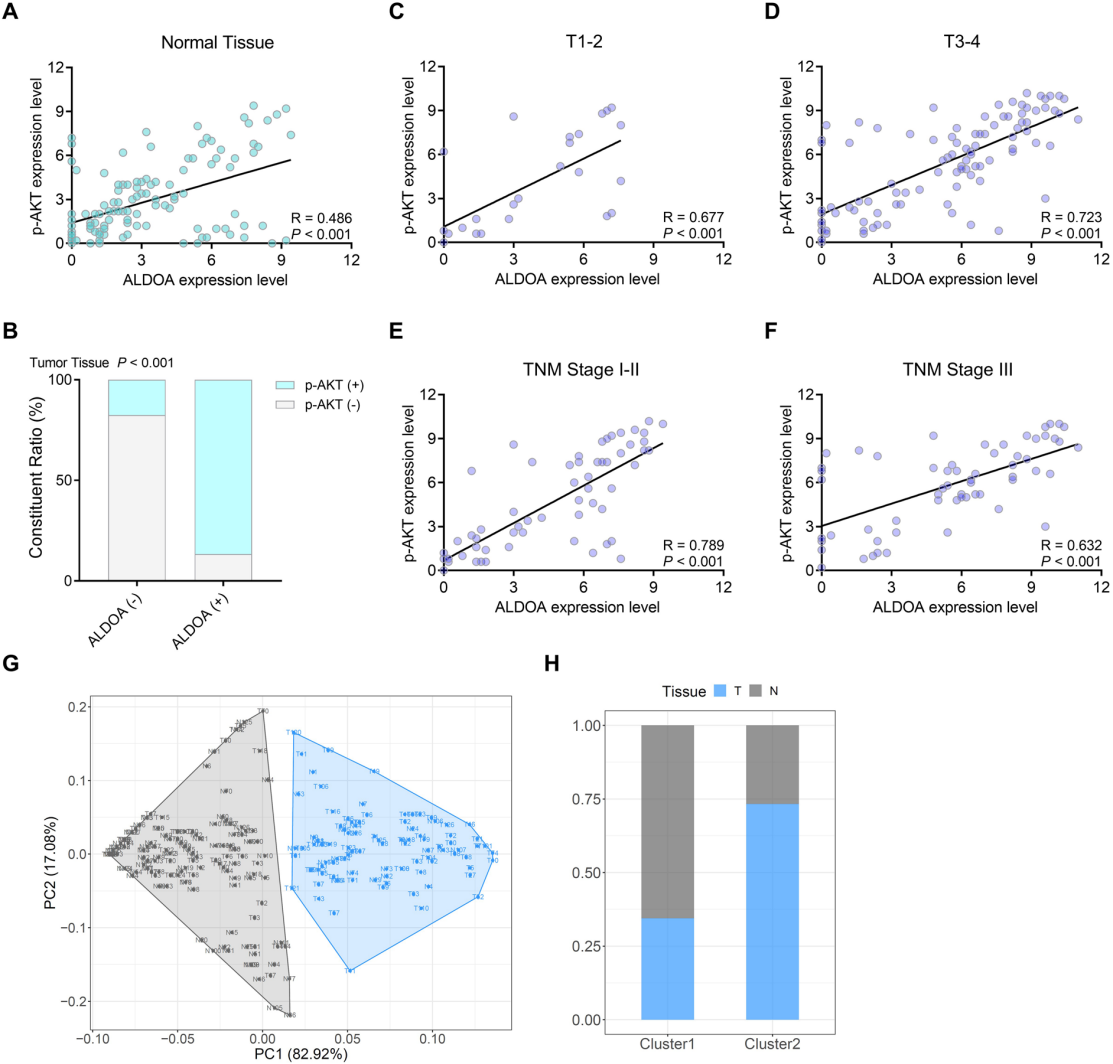
Correlation between ALDOA and p-AKT in CRC and paired normal tissues. (**A**) The association between ALDOA and p-AKT expression according to IHC score in para-cancer normal tissues. (**B**) Constituent ratio displaying the correlation of ALDOA and p-AKT expression in CRC. (**C**-**F**) The association between ALDOA and p-AKT expression according to IHC score in (**C**) the CRC with the T1-2, (**D**) the CRC with the T3-4, (**E**) the CRC with the stage TNM I-II, and (**F**) the CRC with the stage TNM III. (**G**) The stratification of CRC and paired normal tissues in Cluster 1 and Cluster 2 based on the ALDOA and p-AKT expression. (**H**) The percentage of CRC and paired normal tissues in each cluster.

After investigating the association of ALDOA and p-AKT expression in tumor tissue, we also assess the association in normal tissue. Interestingly, in paired normal tissue, ALDOA expression was positively associated with the p-AKT expression (Fig. 4A). The staining results of ALDOA and p-AKT were divided into negative and positive, and constituent ratio displayed the ALDOA and p-AKT expression were also positively correlated in CRC (Fig. 4B). Then the IHC staining was checked to verify the association of ALDOA/p-AKT in subgroup. The outcome suggest that no matter in T1-2 or T3-4 subgroups, the ALDOA expression was significantly correlated with the p-AKT expression in CRC tissues (*P* < 0.001, Fig. 4C,D ). Similarly, in subgroup of various TNM stages, the result indicated that the positive association of ALDOA/p-AKT expression in both TNM I-II and TNM III staging (*P* < 0.001, Fig. 4E,F ). Furthermore, we performed a cluster analysis for normal and tumor tissues based on the expression level of ALDOA/p-AKT in CRC and normal tissues (Fig. 4G), with 41.3% tumor tissues and 78.6% normal tissues in Cluster 1, and 58.7% tumor tissues and 21.4% normal tissues in Cluster 2 (Fig. 4H).

### The influence of ALDOA/p-AKT overexpression on prognosis in CRC patients

**Figure 5:**
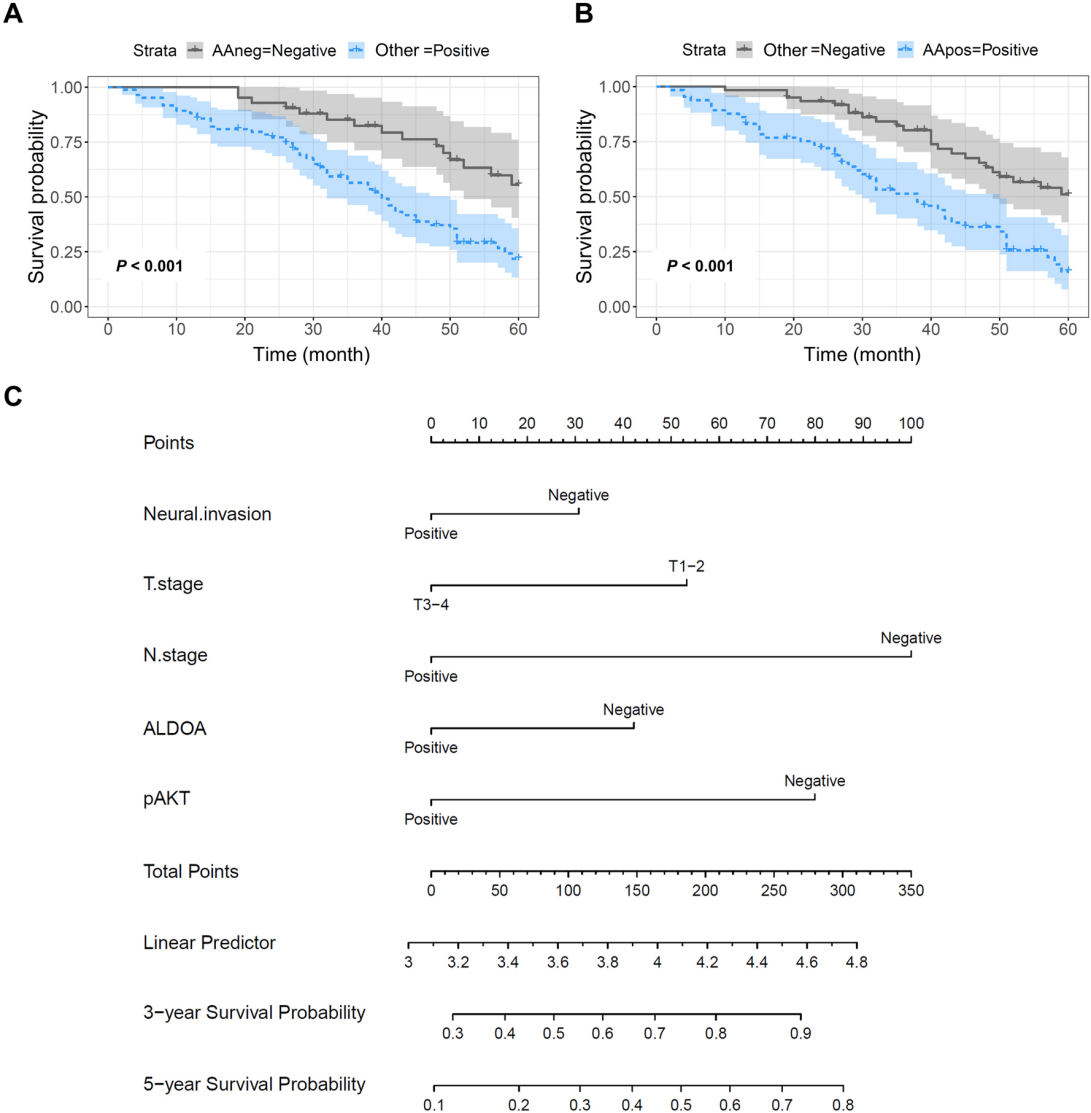
Nomograms predicting 3 and 5-year overall survival of CRC patients. (**A**) Kaplan–Meier analysis of ALDOA/p-AKT negative vs other in 126 CRC patients. (**B**) Kaplan–Meier analysis of ALDOA/p-AKT positive vs other in 126 CRC patients. (**C**) The 3 and 5-year overall survival rate was predicted by the total points which was calculated by each prognostic factor points on the nomogram point scale.

In this study, we found that both ALDOA and p-AKT are significantly elevated in CRC tissue and represent a poor prognosis. In addition, the expression of ALDOA and p-AKT was positively correlated in CRC tissues. To further explore the efficacy of combined ALDOA and p-AKT detection in the prognosis of CRC patients, we first divided 126 CRC patients into ALDOA/p-AKT negative group and other group. The outcome showed the both negative expression of ALDOA/p-AKT group had a better prognosis compared with the other group (*P* < 0.001) (Fig. 5A). Besides, CRC patients were divided into ALDOA/p-AKT positive group and other groups. The both positive expression of ALDOA/p-AKT group had a lower 5-year survival rate compared with the other group (*P* < 0.001) (Fig. 5B).

Furthermore, the nomograms were performed to predict the 3 and 5-year overall survival of CRC patients (Fig. 5C). The survival rate was predicted by the total points which was calculated by each prognostic factor points on the nomogram point scale. The outcome suggested ALDOA/p-AKT expression played crucial roles in predicting the 3 and 5-year overall survival of CRC patients.

## Discussion

CRC is the third most common cancer in the world, and its incidence is rising rapidly in developed countries. Although surgical resection, chemotherapy and other treatment strategies significantly prolong the survival of CRC patients, chemotherapy resistance leads to poor prognosis^2,16^. Therefore, discovering new therapeutic targets and exploring the potential mechanisms are crucial for the treatment of CRC.

ALDOA is mainly expressed in muscle tissue. The ectopic expression of ALDOA plays an important role in the occurrence and development of myocardial hypertrophy, heart failure and many cardiovascular and cerebrovascular diseases^17^. With the further study of ALDOA in recent years, it has been found ALDOA is involved in gluconeogenesis and glycolysis. Both gluconeogenesis and glycolysis can provide energy for tumor proliferation. Metabolic reprogramming can promote tumor cell growth by producing ATP and provide precursors for macromolecule synthesis^3,6,10^. Previous studies have indicated that oncogenes enhance glycolysis by increasing the expression of specific glucose transporters and glycolytic enzymes, promoting the proliferation of cancer cells. Studies have shown that ALDOA is included in the pathological processes of various cancers^9,12,13,18^.

In this study, we found that the expression of ALDOA was significantly elevated in CRC tissues, and ALDOA expression level increased with the progression of the depth of invasion and lymph node metastasis by immunohistochemical evaluation. In survival assessment, high expression of ALDOA predicted a poor prognosis. These findings suggest that evaluating the alteration of ALDOA expression level during the development of CRC has a potential role in predicting disease progression and prognostic outcome. Interestingly, in further analysis, we found that subgroup survival analysis based on TNM stage revealed that increased ALDOA expression level in the TNM I-II staging subgroup represented poor survival, while there was no statistical correlation between ALDOA expression level and survival prognosis in the TNM III staging subgroup. Therefore, if ALDOA expression is used to evaluate the prognosis of CRC patients in clinical applications, the detection of ALDOA expression is more significant in predicting survival for CRC patients with early stage.

ALDOA was found to act as a glycolysis monitor to regulate the NLRP3 inflammasome by sensing changes in the glycolysis process^3^. In this process, ALDOA maintains mitochondrial damage caused by NLRP3 agonists by controlling the AMPK-mTOR signaling pathway. In addition, ALDOA knockout enhanced the AMPK signaling pathway and inhibited mTORC1^3,19^. According to previous studies, and considering the potential role of ALDOA expression in the process of gluconeogenesis and glycolysis of tumor cells, we explored the potential correlation between ALDOA and AKT, another important upstream target of mTOR^20–22^. PI3K-AKT is an important metabolism-related pathway. Studies have also found that AKT phosphorylation levels are significantly elevated in a variety of tumor cells and are potentially correlated with survival prognosis^23–25^.

We evaluated the gene expression correlation between *AKT* and *ALDOA* in the TCGA dataset using the GEPIA platform, and the results showed a positive correlation between the expression of *AKT* and *ALDOA* in colon cancer and rectal cancer tissues. We then further evaluated the correlation between the IHC scores of ALDOA and p-AKT in 126 CRC tissues, and the results showed they were positively correlated in tumor tissues. Interestingly, in paired normal colorectal tissues, the expression of ALDOA and p-AKT were also positively correlated. Based on the above findings, we used ALDOA and p-AKT as factors to construct a CRC survival assessment model, and the results showed that the expression of ALDOA and p-AKT in the prediction model acted as a crucial role.

## Conclusion

According to IHC staining score analysis, the expressions of ALDOA and p-AKT in CRC tissues were remarkably higher than those in adjacent tissues, and aberrant expressions of ALDOA/p-AKT led to poor prognosis in CRC patients. The expression of ALDOA in CRC was remarkably correlated with the expression of p-AKT. Kaplan–Meier analysis revealed that the 5-year survival rate of the group with negative ALDOA/p-AKT was better than that of the other groups. Besides, the both positive expression of ALDOA/p-AKT group had a lower 5-year survival rate compared with the other group. Moreover, the nomograms to predict the 3 and 5-year overall survival of CRC patients showed ALDOA/p-AKT expression played crucial roles in predicting the 3 and 5-year overall survival of CRC patients. Therefore, ALDOA/p-AKT may act as an important role in CRC, which may provide new horizons for targeted therapies.

## Materials and methods

### Patients and tissue specimens

This study included 126 cases of CRC tissue and para-cancer tissues obtained from 2015 to 2016 at the Department of General Surgery, the First Affiliated Hospital of Wannan Medical College. These CRC patients did not undergo preoperative chemoradiotherapy before surgery. The Surgical specimens were fixed in 10% formalin and embedded in paraffin. Everyone has complete clinical data and was available to be followed up. This study was approved by the Independent Ethics Committee (IEC) of the First Affiliated Hospital of Wannan Medical College (IRB number: 202248). All patients included were provided written informed consent. The demographic information of patients was listed in Table S1.

### Immunohistochemistry (IHC)

These CRC tissues were fixed in 10% formalin and embedded in paraffin. Then the paraffin-embedded tissues were serially cut into 5 μm sections. The ALDOA and p-AKT expression in 126 cases of CRC tissue and para-cancer tissues were investigated using IHC. Sections were incubated with anti-ALDOA (1:100, Abcam, ab252953) and anti-Phospho-AKT (1:100, Abcam, ab81283) at 1:100 dilution at the room temperature for two hours. The whole process was visualized via the IHC staining kit (Zhongshan Biotechnology, Beijing, China).

### IHC score assessment

After the IHC staining, five regions were randomly selected for evaluation by two authors. The IHC score was calculated by multiplying staining intensity (0, negative; 1, weak; 2, moderate; and 3, strong) and extent (0, 0–5%; 1, 6–25%; 2, 26–50%; 3, 51–75%; and 4, > 75%). The IHC scores of the five random fields are used to calculate the average, which is the final IHC score for the sample. For the final staining score, 0 was regarded as − ,1 ~ 4 as + , 5 ~ 8 as ++ , 9 ~ 12 as +  +  + . In this study, ++ or +++ was regarded as high expression, and—or + as low expression.

### Statistical analysis

All the data was expressed as the means ± S.D. The statistical analyses were dealt with SPSS 25.0 software (SPSS Inc., Chicago, IL, USA), GraphPad Prism 8 and R programs (version 3.6.1 for Windows, http://cran.r-project.org/). The t-test (unpaired, two-tailed) or Mann–Whitney U test was used to compare means between groups. The Chi-square test was used to assess the IHC results. *P* < 0.05 was regarded significant difference. The authors have confirmed that all methods were carried out in accordance with relevant guidelines and regulations.

### Supplementary Information

Supplementary Information.

## Data Availability

Data are stored by the corresponding author of this paper and are available upon request.
