# Supplementary material for: Prognosis significance and potential association between ALDOA and AKT expression in colorectal cancer
Source: Sci Rep. 2024 Mar 18;14:6488. doi: 10.1038/s41598-024-57209-5 (PMC10948905; doi:10.1038/s41598-024-57209-5)
Supplement: Supplementary file 1 — Supplementary Information. [file 41598_2024_57209_MOESM1_ESM.docx]

Table S1. The demographic information of patients was listed in Table S1.

| Demographic or Characteristic | Patients | | |  |
| --- | --- | --- | --- | --- |
|  |  |  |  |  |
| Age (years) |  |  |  |  |
| ＜65 |  | 77 |  |  |
| ≥65 |  | 49 |  |  |
| Gender |  |  |  |  |
| Male |  | 73 |  |  |
| Female |  | 53 |  |  |
| Tumor size (cm) |  |  |  |  |
| ＜5 |  | 77 |  |  |
| ≥5 |  | 49 |  |  |
| Depth of tumor invasion |  |  |  |  |
| T1-2 |  | 26 |  |  |
| T3-4 |  | 100 |  |  |
| Lymph node metastasis |  |  |  |  |
| No |  | 65 |  |  |
| Yes |  | 61 |  |  |
| Degree of differentiation |  |  |  |  |
| Well |  | 106 |  |  |
| Poor |  | 20 |  |  |
| Venous invasion |  |  |  |  |
| Negative |  | 97 |  |  |
| Positive |  | 29 |  |  |
| Neural invasion |  |  |  |  |
| Negative |  | 97 |  |  |
| Positive |  | 29 |  |  |
| TNM stage |  |  |  |  |
| I-II |  | 65 |  |  |
| III |  | 61 |  |  |
